# Loss of CDKN1B/p27Kip1 expression is associated with ERG fusion-negative prostate cancer, but is unrelated to patient prognosis

**DOI:** 10.3892/ol.2013.1563

**Published:** 2013-09-04

**Authors:** HÜSEYIN SIRMA, MARGARETHE BROEMEL, LAURA STUMM, TINA TSOURLAKIS, STEFAN STEURER, PIERRE TENNSTEDT, GEORG SALOMON, UWE MICHL, ALEXANDER HAESE, RONALD SIMON, GUIDO SAUTER, THORSTEN SCHLOMM, SARAH MINNER

**Affiliations:** 1 Institute of Pathology, Prostate Cancer Center and Section for Translational Prostate Cancer Research at Clinic of Urology, University Medical Center Hamburg-Eppendorf, Hamburg D-20246, Germany; 2 Martini Clinic, Prostate Cancer Center and Section for Translational Prostate Cancer Research at Clinic of Urology, University Medical Center Hamburg-Eppendorf, Hamburg D-20246, Germany

**Keywords:** p27, ERG fusion, prostate cancer

## Abstract

The cyclin-dependent kinase inhibitor p27Kip1 has been suggested as a prognostic marker in prostate cancer. The aim of this study was to determine the clinical and prognostic role of p27 expression in hormone-naive prostate cancers. A tissue microarray containing samples from 4,699 prostate cancers with attached pathological, clinical follow-up and molecular data was analyzed for nuclear p27 expression by immunohistochemistry. p27 staining was negative in 18.6%, weak in 33.5%, moderate in 28.4% and strong in 19.5% of 3,701 interpretable cancer spots. Loss of p27 immunostaining was linked to tumors of low Gleason grade (P<0.0001) and ERG fusion-negative cancers (P<0.0001). p27 levels were not associated with other parameters, including tumor stage, nodal stage, preoperative prostate-specific antigen (PSA) levels, surgical margin status and cell proliferation (as measured by the Ki67 labeling index). p27 expression was also unrelated to clinical outcome in all cancers, as well as in the subsets of ERG fusion-positive and -negative cancers. Overall, the present data demonstrated that elevated p27 expression was often unrelated to prostate cancer phenotype. Furthermore, the lack of an effect of the p27 protein levels on PSA recurrence following radical prostatectomy indicated that factors other than p27 expression are likely to be the major determinants of prostate cancer recurrence. However, a subset of ERG-negative, low-grade tumors was frequently characterized by loss of p27, suggesting a role of this alteration for the development of these tumors.

## Introduction

Prostate cancer is a significant health problem and a leading cause of cancer-associated mortality in males. Approximately 50% of males develop prostate cancer during their lifetime, and about half of these cancers become potential life-threatening disease requiring therapeutic intervention (1). Thus, molecular markers discriminating between indolent and aggressive forms of the disease are highly desired but currently lacking (2,3).

The expression levels of critical cell cycle regulators, such as cyclin dependent kinases (CDK) and their inhibitors, are frequently altered in cancers. The CDK inhibitor p27Kip1 has often been proposed as a prognostic marker in human cancers (4). p27 inhibits CDK2 and CDK4 and induces growth arrest. The observed deregulation of p27 expression in cancers is not primarily due to mutations, but is instead caused by ubiquitin-mediated proteasomal degradation (4). Reduced levels of p27 occur in several cancer types and are generally associated with poor prognoses. For example, loss of p27 has been revealed to be an independent prognostic factor in breast, colon and gastric carcinomas (4,5). Studies investigating the prognostic significance of p27 in prostate cancer have yielded conflicting results [reviewed in (6)]. A number of studies have proposed that tumors with lost or diminished p27 expression are more aggressive (7–16), while other studies could not confirm these data (17–22). As all previous studies on the prognostic relevance of p27 expression in prostate cancer have analyzed comparatively small tumor sets with a maximum of 130 patients, it is possible that the analysis of large cohorts may provide clearer results.

In the present study, a large tissue microarray (TMA) containing samples from 4,699 hormone naive prostate cancers, obtained from patients who had undergone radical prostatectomy, was used. The present data showed that the loss of p27 expression was correlated with ERG fusion-negative tumors, but did not identify a significant effect of p27 expression on prostate cancer phenotype or patient prognosis.

## Materials and methods

### Patients

A set of prostate cancer prognosis TMAs containing one 0.6-mm tissue core each from 4,699 consecutive radical prostatectomy specimens, obtained from patients undergoing surgery between 1992 and 2008 at the Department of Urology or the Martini Clinic at the University Medical Center Hamburg-Eppendorf (Hamburg, Germany), was used in the present study. The pathological and clinical data of the arrayed prostate cancers are shown in Table I. The composition of this TMA is described in detail in Table II. Clinical follow-up data were available for 4,203 patients and the median follow-up was 46.7 months, ranging between one and 219 months. None of the patients received neoadjuvant or adjuvant therapy prior to prostate-specific antigen (PSA) recurrence, which was the clinical endpoint of this study. The first PSA value ≥0.2 ng/ml following surgery was used to define the time of recurrence. Fluorescence *in situ* hybridization (FISH) data for ERG and immunohistochemical ERG (23) and Ki67 (24) results were available from previous studies. This study was approved by the ethics committee of the University Medical Center Hamburg-Eppendorf (Hamburg, Germany). Written informed consent was obtained from the patients.

### Immunohistochemistry (IHC)

Freshly cut TMA sections were used for immunostaining. Slides were deparaffinized and exposed to heat-induced antigen retrieval for 5 min in an autoclave at 121°C and pH 9.0. p27 IHC was performed using a monoclonal antibody (1:50; DCS72; Calbiochem, Darmstadt, Germany). An EnVision™ system (Dako, Glostrup, Denmark) was used to visualize the immunostaining. Nuclear p27 staining was evaluated according to the following scoring system. The staining intensity (0, 1+, 2+ and 3+) and the fraction of positive tumor cells were recorded for each tissue spot. A final score was built from these two parameters as follows: Negative, staining intensity of 0; weak, staining intensity of 1+ in ≤70% of tumor cells or staining intensity of 2+ in ≤30% of tumor cells; moderate, staining intensity of 1+ in >70% of tumor cells, staining intensity of 2+ in >30% but ≤70% of tumor cells or staining intensity of 3+ in ≤30% of tumor cells; strong, staining intensity of 2+ in >70% of tumor cells or staining intensity of 3+ in >30% of tumor cells.

### Statistical analysis

For the statistical analysis, JMP 8.0 software (SAS Institute, Inc., Cary, NC, USA) was used. Contingency tables were calculated to study associations between the p27 score and clinico-pathological variables. The χ^2^ (likelihood ratio) test was used to identify significant associations. Kaplan-Meier curves were generated for PSA recurrence-free survival. The log-rank test was applied to test the significance of differences between stratified survival functions. Cox proportional hazards regression analysis was performed to test the statistical independence and significance between pathological, molecular and clinical variables.

## Results

### Technical issues

A total of 4,699 hormone-naive cancers were analyzed for p27 expression. The analysis was successful in 3,701 tumors and failed in 998 cases (21.2%) due to lack of tissue spots or absence of unequivocal cancer cells in the p27-stained TMA section. Data were available for genomic ERG rearrangement (by FISH; n=2,336), ERG expression (by IHC; n=4,266) and Ki67 labeling index (by IHC; n=3,711) from previous studies (22,23).

### p27 expression in prostate cancer

**Figure 1:**
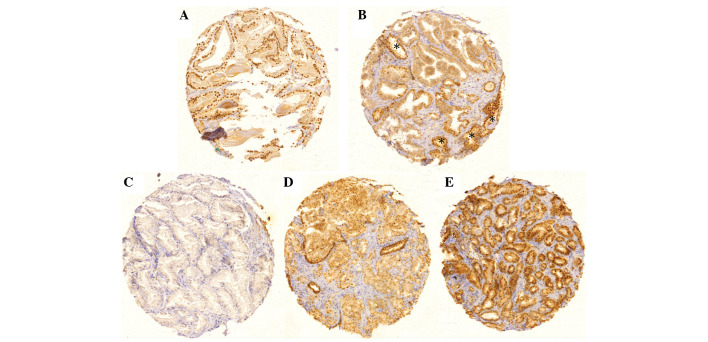
Representative images of p27 immunostaining. (A) Positive nuclear staining in a spot harboring non-neoplastic prostate epithelium; (B) loss of p27 staining in neoplastic epithelium, but not in normal epithelium (*); (C) negative staining in prostate cancer; (D) moderate positive staining in prostate cancer; (E) strong positive staining in prostate cancer.

**Figure 2:**
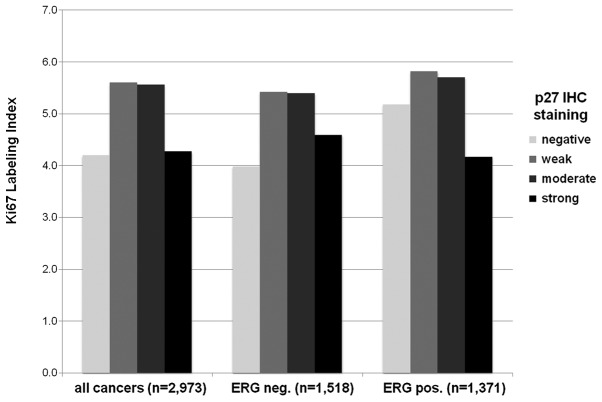
Association of p27 staining score with Ki67 labeling index (Ki67Li) in all prostate cancers, and in ERG fusion-negative (ERG neg.) and -positive (ERG pos.) prostate cancers.

Examples of p27 immunostaining are shown in Fig. 1. Strong p27 expression was observed in tissue spots containing normal prostate tissue and was predominantly identified in the nucleus, with weak diffuse cytoplasmic staining, and was almost undetectable in stromal cells. By contrast, negative or weak nuclear p27 expression was observed in 52.1% of the 3,701 interpretable tumor spots. Expression was categorized as negative in 18.6% (n=689), weak in 33.5% (n=1,239), moderate in 28.4% (n=1,052) and strong in 19.5% (n=721) of the tumor spots analyzed. Associations between the p27 expression score and tumor phenotype are shown in Table II. Loss of p27 was correlated with low Gleason grade; there was a decrease in the fraction of p27-negative tumors from Gleason ≤3+3 (25.5%) to Gleason ≥4+4 (8.3%) (P<0.0001). This was paralleled by an increase in the fraction of tumors with strong p27 staining from 16.5% in Gleason ≤3+3 to 24.1% in Gleason ≥4+4. All data are summarized in Table II. The levels of p27 staining were unrelated to other parameters, including nodal stage, surgical margin status and cell proliferation index as measured by the Ki67 labeling index (Fig. 2). A significant P-value was observed for the association between the p27 score and tumor stage (P=0.0185). However, we did not consider this association to be true, as the fraction of tumors with negative, weak, moderate or strong p27 scores was almost identical in the different tumor stages.

### Association of p27 with ERG gene breaks and ERG protein expression, and the clinical significance of this

**Figure 3:**
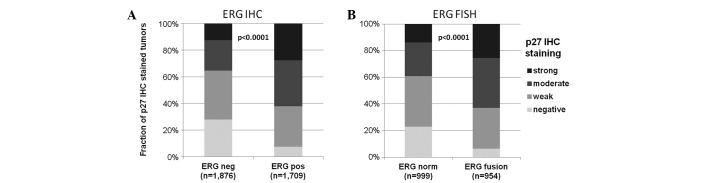
Association of p27 immunohistochemical staining and ERG fusion status. Comparison of p27 staining scores in ERG-positive and -negative prostate cancers. ERG fusion status was determined by either (A) immunohistochemistry (IHC) or (B) fluorescence *in situ* hybridization (FISH).

**Figure 4:**
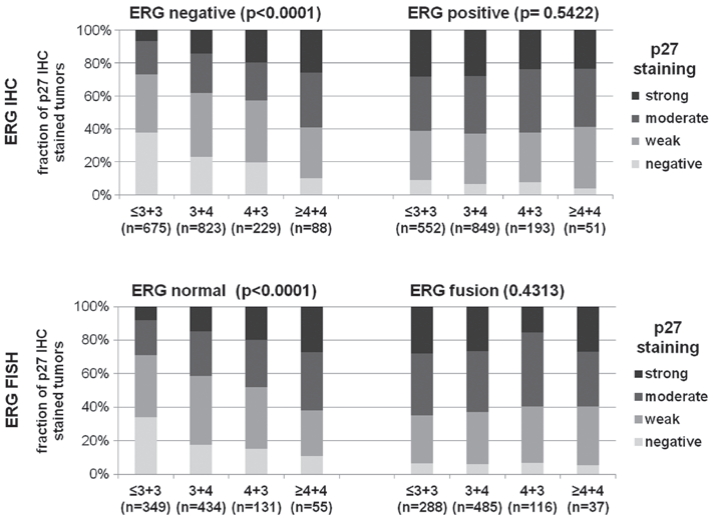
Association of p27 immunohistochemical staining and Gleason grade in ERG fusion-negative and -positive prostate cancers by immunohistochemistry (IHC) and fluorescence *in situ* hybridization (FISH) analysis.

The availability of ERG data from a previous study (23) made it possible to search for associations between ERG and p27, and compare p27 scores and prostate cancer phenotypes, in separate subsets of ERG fusion-positive and -negative cancers. Loss of p27 staining was associated with ERG fusion-negative cancers. This was true for immunohistochemical detection of ERG protein expression and FISH detection of ERG breaks (P<0.0001 for both; Fig. 3). Loss of p27 expression was observed in 7.6% of all ERG fusion-positive tumors compared with 27.8% in ERG fusion-negative tumors, as revealed by ERG IHC (Fig. 3A). Consistent with this result, p27 expression was considered to be negative in only 6.5% of ERG fusion-positive cancers compared with 22.8% in ERG fusion-negative cancers, as revealed by ERG FISH (Fig. 3B). Notably, subset analysis showed that the loss of p27 was correlated with low Gleason grades only in ERG fusion-negative cancers (P<0.0001) and not in ERG-positive tumors (P=0.5422, Fig. 4) by IHC. This significant association in ERG fusion-negative tumors was demonstrated by FISH analysis of ERG rearrangement (P<0.0001; Fig. 4). Other parameters did not show significant associations in ERG-negative or -positive cancers, including tumor stage, nodal stage and surgical margin status (data not shown).

**Figure 5:**
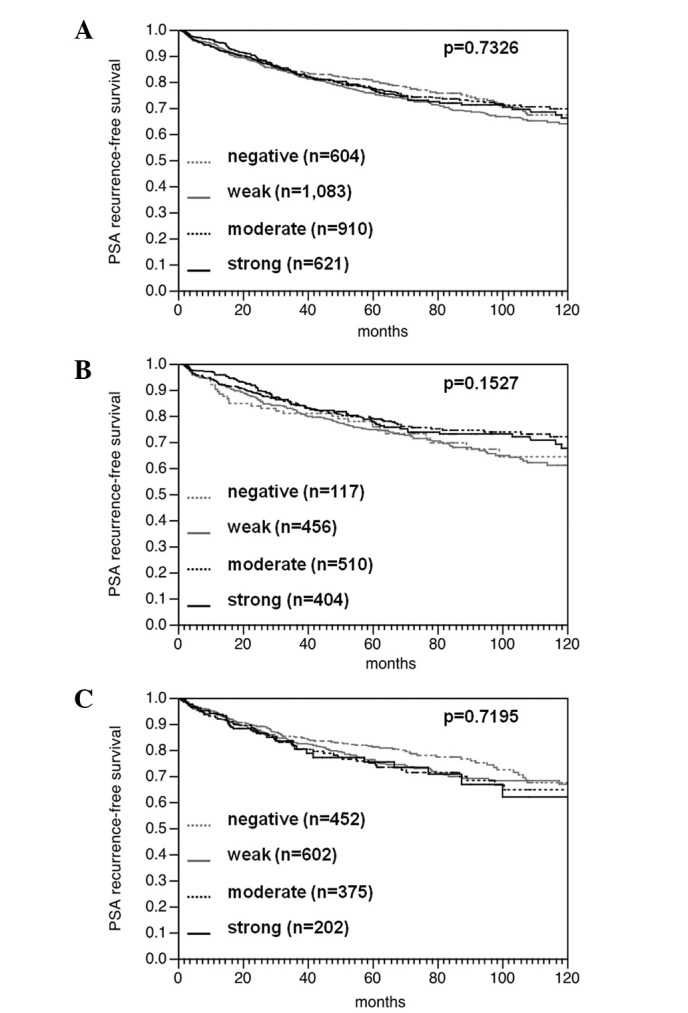
Prostate-specific antigen (PSA) recurrence-free survival stratified for p27 staining score in (A) all prostate cancers, as compared with subsets of (B) ERG fusion-negative and (C) ERG fusion-positive prostate cancers.

No associations were observed between the various p27 staining scores and patient prognosis in all cancers, nor in the subsets of ERG fusion-positive or -negative cancers (Fig. 5).

## Discussion

The prognostic value of p27 expression in prostate cancer has been discussed with contrary conclusions in a number of previous studies [reviewed in (6)]. In the present study, >4,000 prostate cancers with long-term clinical data were analyzed in a TMA format. This database enabled assessment of the effect of p27 separately and in the two major molecular subgroups of prostate cancer, as defined by the ERG fusion status. The data revealed negative and weak p27 staining scores in 18.6 and 33.5% of samples, respectively, and showed that the loss of p27 expression was associated with ERG-negative cancers. The frequency of tumors with reduced p27 expression was within the range of previous studies, which reported absent or reduced p27 expression in 16–68% of cancers (6,12,13). The present results clearly demonstrated that the loss of p27 expression had no prognostic significance in radically operated prostate cancers. In particular, there was no clear difference with respect to PSA recurrence between strongly p27-positive and -negative cancers, neither when tumors were jointly analyzed, nor in subsets of ERG fusion-negative and -positive cancers. This lack of prognostic relevance is concordant with data from a number of other studies that were also unable to identify a prognostic effect of reduced p27 expression (17–22). Other studies have demonstrated that prostate cancers with a loss of or diminished p27 expression have particularly poor prognoses (7–11,13–15,17,25). It is possible that these controversial data are partly attributable to sampling issues, as these studies have all analyzed relatively small patient sets comprising between 86 and up to 130 prostate cancers. The present data, together with published findings, clearly argue against a clinically relevant impact of p27 expression on prostate cancer development and progression. This hypothesis is also consistent with the lack of association between p27 expression and tumor cell proliferation, as well as the observation that p27 knockout results in only a moderate increase in prostate epithelial proliferation and altered differentiation (15,26).

In the present study, the loss of p27 expression was significantly associated with ERG fusion-negative prostate cancers. The association with ERG was demonstrated by two independent approaches, namely IHC and FISH. This finding largely excluded the possibility of an artificial association caused by false negative IHC for both parameters in a subset of tissues that may have tissue damage compromising immunoreactivity. Previous studies have already demonstrated that there are considerable molecular differences between ERG fusion-positive and -negative prostate cancers (27–29). A number of studies have demonstrated gene expression profiles that were markedly distinctive between ERG fusion-positive and -negative cancers (30–32). At the level of specific genes and pathways, it is now well accepted that ERG interacts and modulates several tumor-relevant pathways involving AR, C-MYC, NKX3.1 and PTEN, all of which are more frequently identified to be altered in ERG fusion-positive prostate cancer, compared with ERG fusion-negative prostate cancer (27,28,33). The present study revealed that the loss of p27 protein expression is another feature associated with ERG-negative tumors.

In the present study, loss of p27 was associated with tumors of low Gleason grade. This is in contrast to numerous earlier studies that either did not identify a significant correlation (10,11,14,18,21,22) or even reported inverse associations (7,8,12,16,19,20,25). Of note, the association with low-grade cancers was limited to the subset of ERG fusion-negative tumors. This finding suggests a distinct role for p27 loss in ERG fusion-positive and -negative cancers. In ERG fusion-positive cancers, the fraction of tumors with p27 loss was low and unrelated to the Gleason grade, suggesting that neither tumor differentiation nor progression are dependent on p27 levels. By contrast, in ERG fusion-negative cancers, there was a noticeably large fraction (40%) of low-grade cancers harboring p27 loss. This fraction significantly decreased to ~10% in high-grade (Gleason ≥4+4) cancers. It may be hypothesized that the loss of p27 favors the development of cancers that are characterized by low malignant potential.

**Figure 6:**
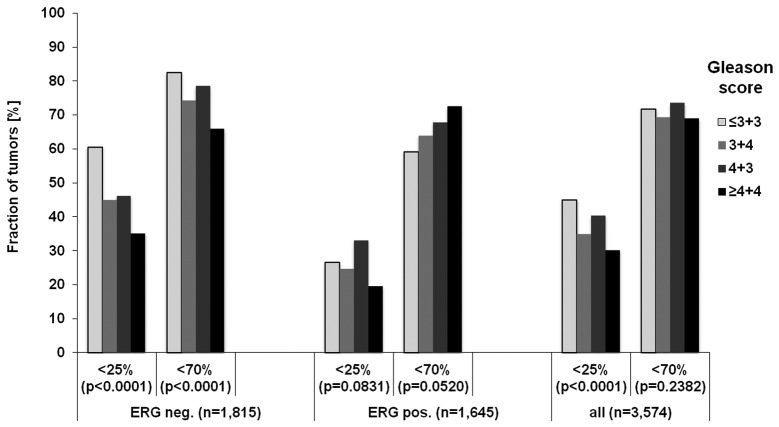
Association of p27 loss and Gleason grade in ERG fusion-negative (ERG neg.) and -positive (ERG pos.) prostate cancers, as well as in all prostate cancers, according to the criteria used by Tsihlias *et al* (<25%) and Vlachostergios *et al* (<70%) (6,20).

The majority of earlier studies analyzing p27 in prostate cancer were performed prior to the discovery of ERG fusions in 2005 (33), and later studies did not consider the ERG status (20–22). It is possible that variable fractions of ERG fusion-negative or -positive cancers may obscure this association, particularly if only small sample sizes are analyzed. In addition, it cannot be excluded that the various criteria applied to define loss or low p27 expression may have a significant effect on the outcome in different studies. For example, Vlachostergios *et al* observed reduced p27 expression in 86% of cancers defined as <70% of tumor cells staining positive for p27 (21). By contrast, Tsihlias *et al* defined low expression as <25% of tumor cells staining positive for p27 (7). To estimate the effect of different scoring systems on the associations tested, these scores were rebuilt in the present dataset according to the criteria used by Vlachostergios *et al*(21) and Tsihlias *et al*(7). Both positive and negative associations between the loss of p27 and Gleason grades based on the ERG status, as well as the scoring system used, were observed (Fig. 6). In the present study, an established scoring system based on the staining intensity and the fraction of tumor cells, which has been successfully used in numerous previous studies, was used to detect associations between molecular markers and tumor phenotype or patient prognosis (34,35). Such a predefined scoring system has the advantage that it includes the important information of staining intensity and enables an unbiased, standardized analysis. However it may not be optimally suited for establishing potential diagnostic thresholds.

The present data also demonstrated the power of large-scale TMAs for the evaluation of biomarkers. Although the use of just one 0.6-mm TMA spot per donor tissue typically results in a loss of 20–30% of data points due to lack of cancer in individual TMA spots, the number of interpretable tissues remains sufficient for high-power statistical analysis in large TMAs. The ‘one core per donor tissue’ approach has the important advantage that the same amount of tissue (one spot) is analyzed per patient. TMAs containing two or more samples per tumor suffer from the statistical problem whereby individual samples are not always interpretable, resulting in patient subgroups with one, two, three and perhaps more interpretable samples, which in turn leads to higher positivity rates in patients with more interpretable tissue samples (36). In various earlier studies, large-scale prostate cancer TMAs that included >3,000 patients were used to demonstrate the prognostic role of p53, HER2, EGFR, Ki67LI, 8p deletion and PSMA (23,24,35,37–39). The large number of cases included in prostate cancer TMAs increasingly enables the selective analysis of relevant, molecularly defined subsets, such as ERG-positive prostate cancers.

In summary, the results of the present study have demonstrated that p27 expression was lost in a considerable fraction (~20%) of prostate cancers, but had no overall effect on patient prognosis and cancer progression. Differences may exist, however, in the biology of p27 in ERG fusion-negative and -positive cancers.

## Figures and Tables

**Table I:** Pathological and clinical data of the arrayed prostate cancers.

	No. of patients	
	
Data	Study cohort on TMA (n=4699)	Biochemical relapse among categories (n=904)
Follow-up (months)		
Mean	56.6	-
Median	46.7	-
Age (years)		
<50	126	22
50–60	1399	227
61–70	2596	520
>70	337	84
Pretreatment PSA (ng/ml)		
<4	666	77
4–10	2559	376
10–20	894	269
>20	289	159
pT category (AJCC 2002)		
pT2	3010	272
pT3a	926	286
pT3b	489	307
pT4	42	39
Gleason grade		
≤3+3	1761	125
3+4	2055	450
4+3	512	261
≥4+4	135	68
pN category		
pN0	2317	580
pN+	151	111
Surgical margin		
Negative	3634	573
Positive	810	324

Numbers do not always add up to 4,699 in the various categories due to cases with missing data. TMA, tissue microarray; PSA, prostate-specific antigen; pT, primary tumor; AJCC, American Joint Committee on Cancer; pN, regional lymph node.

**Table II:** Clinicopathological features of study cohort, associations with p27 IHC and subsets of ERG-negative and -positive cancers.

			p27 IHC result (all cancers/ERG-negative/ERG-positive)				
				
Parameter	n all	n evaluable	Negative (%)	Weak (%)	Moderate (%)	Strong (%)	P-value
All cancers	4699	3701 (1876/1709)	18.6 (27.8/7.5)	33.5 (36.9/30.3)	28.4 (22.9/34.7)	19.5 (12.4/27.5)	
Tumor stage							
pT2	3019	2338 (1260/994)	20.1 (29.5/7.0)	32.9 (36.0/29.5)	27.9 (22.6/34.7)	19.2 (11.9/28.8)	0.0185 (0.0758/0.2009)
pT3a	946	778 (342/420)	15.8 (23.1/8.1)	33.3 (39.8/28.8)	29.1 (22.0/35.2)	21.9 (15.2/27.9)	
≥pT3b	527	461 (216/231)	15.0 (22.2/8.7)	37.5 (38.0/37.2)	29.5 (26.4/32.0)	18.0 (13.4/22.1)	
Gleason grade							
≤3+3	1765	1279 (675/552)	25.5 (37.9/9.2)	32.1 (35.1/29.7)	25.9 (20.3/32.6)	16.5 (6.7/28.4)	<0.0001 (<0.0001/0.5422)
3+4	2074	1715 (823/894)	15.0 (23.0/6.6)	34.5 (38.9/30.5)	29.3 (23.9/34.9)	21.2 (14.2/28.0)	
4+3	528	435 (229/193)	14.9 (19.7/7.8)	34.0 (37.6/30.1)	30.1 (23.1/38.3)	20.9 (19.7/23.8)	
≥4+4	166	145 (88/51)	8.3 (10.2/3.9)	33.1 (30.7/37.3)	34.5 (33.0/35.3)	24.1 (26.1/23.5)	
Lymph node metastasis							
N0	2359	1837 (916/877)	16.2 (23.9/7.4)	34.0 (37.7/30.7)	29.5 (24.5/34.9)	20.3 (14.0/27.0)	0.9015 (0.1647/0.6316)
N+	174	150 (73/73)	14.0 (17.8/8.2)	34.0 (32.9/35.6)	30.7 (26.0/35.6)	21.3 (23.3/20.6)	
Preoperative PSA level (ng/ml)							
<4	672	493 (217/247)	17.0 (26.7/7.7)	30.0 (33.2/27.1)	31.9 (26.3/36.4)	21.1 (13.8/28.8)	0.002 (0.1787/0.0023)
4–10	2585	2060 (1039/964)	17.9 (27.6/6.2)	33.7 (38.0/30.0)	27.3 (20.1/34.1)	21.0 (13.8/29.8)	
10–20	909	734 (413/303)	21.4 (30.0/9.2)	33.8 (35.6/31.0)	30.0 (24.7/38.3)	14.9 (9.7/21.5)	
>20	311	249 (134/107)	18.1 (20.9/13.1)	39.8 (39.6/41.1)	24.1 (26.1/21.5)	18.1 (13.4/24.3)	
Surgical margin							
Negative	3665	2866 (1473/1299)	18.5 (27.5/7.3)	33.7 (37.1/30.3)	27.9 (22.9/34.0)	19.8 (12.6/28.5)	0.6104 (0.9984/0.3255)
Positive	844	687 (328/340)	18.5 (27.5/8.5)	32.9 (36.9/30.6)	30.3 (22.8/37.1)	18.3 (12.8/23.9)	

IHC, immunohistochemistry; PSA, prostate-specific antigen.
